# Influence of Different Parenting Styles on Child’s Behaviour in a Dental Operatory and Dental Caries Experience: A Systematic Review and Meta‐Analysis

**DOI:** 10.1155/ijod/9390631

**Published:** 2026-09-15

**Authors:** Gauri Kalra, Apurva Mishra, Kusum Bharti, Vijay Prakash Mathur

**Affiliations:** ^1^ Department of Pediatric and Preventive Dentistry, Manav Rachna Dental College, Faridabad, Haryana, India, mrdc.ac.in; ^2^ Department of Clinical Sciences, College of Dentistry, Ajman University, Ajman, UAE, ajman.ac.ae; ^3^ Center of Medical and Bio-Allied Health Sciences Research, Ajman University, Ajman, UAE, ajman.ac.ae; ^4^ Department of Pediatric and Preventive Dentistry, Post Graduate Institute of Child Health, Noida, Uttar Pradesh, India; ^5^ Department of Pediatric and Preventive Dentistry, Centre for Dental Education and Research, AIIMS, New Delhi, India, aiims.edu

**Keywords:** child behaviour, dental anxiety, dental caries, parent–child interaction

## Abstract

**Background:**

Parental influence plays a critical role in shaping a child’s behaviour during dental visits, particularly when parents themselves have experienced negative dental encounters. This systematic review investigated the association between parenting styles (PSs), children’s behaviour in dental settings and dental caries (DC) experience.

**Methods:**

This systematic review followed the Preferred Reporting Items for Systematic Reviews and Meta‐Analyses (PRISMA 2020) and was registered in PROSPERO (CRD42024550441). Electronic databases and grey‐literature sources were searched for studies published from January 2014 to December 2025. Risk of bias was assessed using the revised Joanna Briggs Institute critical appraisal tool for analytical cross‐sectional studies. Extractable dental‐behaviour data were pooled using generic inverse‐variance random‐effects models with restricted maximum‐likelihood estimation and modified Hartung–Knapp confidence intervals. DC outcomes were synthesised narratively because of clinical and methodological heterogeneity. Certainty of evidence was assessed using GRADE.

**Results:**

Seventeen cross‐sectional studies met the eligibility criteria. Thirteen studies (76.5%) included only children aged up to 6 years, and the Parenting Styles and Dimensions Questionnaire (PSDQ) was used in 13 studies (76.5%). Twelve studies assessed dental behaviour or cooperation and 12 assessed DC; seven evaluated both outcomes. In the primary meta‐analysis, authoritative parenting was associated with higher odds of positive dental behaviour than permissive parenting (OR 4.77, 95% CI 1.10–20.76; *I* ^2^ = 88.1%) and authoritarian parenting (OR 7.95, 95% CI 2.32–27.29; *I* ^2^ = 54.1%). The 95% prediction intervals for both comparisons crossed the null. No clear difference was found between permissive and authoritarian parenting (OR 0.87, 95% CI 0.36–2.10; *I* ^2^ = 19.2%). DC findings were inconsistent. Certainty of evidence was very low for all three behavioural comparisons.

**Conclusion:**

Authoritative parenting was associated with more positive dental behaviour than permissive or authoritarian parenting. However, the cross‐sectional design, residual confounding, heterogeneity, wide prediction intervals and very low‐certainty evidence preclude causal interpretation. Evidence for DC was mixed and did not support a pooled estimate.

## 1. Background

Children’s reactions vary greatly during dental visits and are affected by factors such as age, health, cognitive level, cultural background, level of anxiety or fear, social expectations, comfort around strangers and their own personality traits [1]. Consequently, their behaviour during dental visits may be difficult to predict [2]. Parents play a crucial role in modifying the child’s behaviour during dental visits, particularly if they have had past negative experiences. Their involvement also helps in guiding the child’s long‐term oral health habits. Several researchers have shown an association between parents’ oral health attitudes and practices and their children’s oral health outcomes [3–6]. The approach and attitude adopted by parents for raising their children, known as parenting style (PS), significantly influences the child’s development surrounding the home environment [7, 8]. Literature has described four types of PSs—authoritarian, authoritative, permissive, and neglectful/uninvolved [7, 9, 10].

Parents who are strict and enforce harsh discipline to mould their child’s behaviour and expect them to be obedient are called authoritarian parents, whereas authoritative parents are warm and supportive and have defined boundaries for their child’s actions. Permissive parents are lenient, exerting minimal control towards the child’s impulses and actions and allowing children to act freely by offering little guidance and support. Neglectful parents provide minimal behavioural guidance and provide the least emotional involvement [11]. Uninvolved parenting is marked by detachment, limited communication, and setting few expectations for the child [12]. Saadia and Valencia [13] described various challenges faced by paediatric dentists while communicating with children and parents, especially with evolving behaviour and attitudes of modern generations.

Dental caries (DC), the most widespread disease affecting children worldwide, is influenced by several factors and significantly affects a child’s well‐being. DC is a multifactorial disease influenced by biological, behavioural and socioeconomic factors [14–16]. Stress and emotional conditions, like fear and personality traits, are all contributing factors that influence children’s behaviours at the dental visit and often making dental treatment challenging to all involved—dentists, parents and children. In addition to child‐related factors such as dental anxiety and temperament, factors related to parents and dentists could also contribute to behavioural problems [17, 18]. Interestingly, these factors often interact with each other. Within families, factors such as parental approaches and attachment style have also been associated with the severity of anxiety in children [19]. Several studies have shown potential relationships between PS, child’s behaviour and caries, but they are not enough to conclusively draw firm conclusions about the nature and strength of these associations [1, 20, 21].

To address the gap in literature pertaining to the scarcity of evidence in association among PS, child’s behaviour and caries, this systematic review has been designed. This review therefore aimed to assess the association of PSs with children’s behaviour in the dental operatory and DC experience.

## 2. Methods

### 2.1. Study Design

This systematic review was conducted and reported in accordance with the Preferred Reporting Items for Systematic Reviews and Meta‐Analyses (PRISMA) 2020 statement and registered in the International Prospective Register of Systematic Reviews (PROSPERO; CRD42024550441) (Supporting Information 1: File S1) [22]. Studies published from January 2014 to December 2025 were eligible. The review question was: What is the association between PS, children’s behaviour in the dental operatory and DC experience?

The PICO components of the research question are as follows:

**Table:** 

P (Population)	Children aged 2–12 years
I or E (Exposure)	Parenting styles classified according to a recognised parenting framework (authoritative, authoritarian, permissive, neglectful)
C (Compare)	Authoritative, authoritarian, permissive and neglectful/uninvolved parenting styles
O (Outcome)	(a) Child’s behaviour in dental operatory (b) Dental caries (DC) status

### 2.2. Search Strategy

Electronic databases such as PubMed, Web of Science, Scopus, Embase and Cochrane were searched using text words and MeSH terms. An additional search was performed using Google Scholar and Open Grey. Three authors independently searched the databases. The broad‐based search was implemented individually with keywords: ‘Parenting style’, “Parenting”, ‘Parent‐Child Relationship’, ‘Child behavior’, ‘Dental caries’, ‘Dental visit’. Partial searches with the Boolean tools ’AND’ and ’OR’ were done with the above keywords individually and with different possible combinations. The detailed search strategy can be found in Supporting Information 2: File S2.

### 2.3. Study Selection

Three authors carried out an independent screening of the titles and abstracts of all the identified articles to evaluate their suitability of inclusion, and duplicates were removed by means of EndNote (EndNoteTMX9, Clarivate Analytics, USA) reference management software. The reference lists of eligible studies were cross‐checked to identify any additional studies.

The following eligibility criteria were chosen for screening of the identified studies:

Inclusion criteria

• Studies included children aged 2–12 years. For the behavioural synthesis, children had to be attending their first dental visit or have no previous dental treatment experience.
• Cross‐sectional studies examine PS in relation to dental‐operatory behaviour and/or DC.
• Studies published between 2014 and 2025.
• Full‐text articles published in English.

Exclusion criteria

• Case reports, case control studies, case series, letter to the editor, comprehensive and systematic reviews.
• For the behavioural synthesis, studies were excluded when participants had previous dental treatment experience, active dental pain, dental or medical phobia.
• Studies involving children with medical conditions limiting cognitive development.

The age cutoff of upto 12 years was selected as it corresponds to the mixed dentition stage, during which parental influence on behaviour and oral health practices is most pronounced. Twelve years literature search timeframe was chosen to capture methodologically consistent studies using validated tools while reflecting contemporary parenting practices and paediatric dental care.

### 2.4. Data Extraction

Three reviewers independently extracted data using a prespecified Microsoft Excel form (version 16.17). Extracted variables included study identification, design and setting, participant characteristics, sampling methods, parenting‐style instrument and distribution, behaviour and caries measures, examiner calibration, outcome data, and study conclusions. For the behavioural meta‐analysis, positive and negative event counts were extracted for each PS group. Any disagreements during data extraction and analysis were resolved through discussion.

### 2.5. Risk of Bias Assessment

Two reviewers independently assessed the risk of bias of the included studies using the revised JBI critical appraisal tool for analytical cross‐sectional studies (2026) [23]. The tool comprises eight items addressing participant selection, measurement of eligibility conditions, validity and reliability of exposure and outcome measurements, identification and management of confounding, appropriateness of statistical analyses, and reporting of participants and study settings. Each item was rated as Yes, No, Unclear, or Not applicable. Disagreements were resolved through discussion and, when required, consultation with a third reviewer. No numerical score or low‐, moderate‐ or high‐risk classification was applied. Item‐level judgements were considered when interpreting the findings.

### 2.6. Data Synthesis, Analysis and Grading

Quantitative synthesis was undertaken for studies reporting extractable numbers of children with positive and negative dental behaviour according to authoritative, authoritarian and permissive PSs. The primary synthesis was restricted to children attending their first dental visit or with no previous dental treatment experience and to studies whose behaviour categories could be mapped unambiguously to a common binary outcome. Frankl ratings 3 and 4 were classified as positive/cooperative behaviour, whereas ratings 1 and 2 were classified as negative/uncooperative behaviour. Equivalent study‐specific categories were harmonised only when their correspondence with these definitions was clear. Unadjusted 2 × 2 event counts were used, and an OR greater than 1 favoured the first PS named in each comparison. A continuity correction of 0.5 was added to all four cells when a study contained a single zero cell; double‐zero comparisons were excluded from relative‐effect estimation.

Random‐effects models were specified a priori because clinical and methodological differences were expected across countries, settings, parenting‐style instruments and behaviour‐assessment procedures. Log Ors were pooled using generic inverse variance weighting. Between‐study variance was estimated using restricted maximum likelihood, and modified Hartung–Knapp 95% CI was calculated to account for the small number of studies [24]. Heterogeneity was assessed using Cochran’s *Q*, *I* ^2^ and *τ* ^2^, and a 95% prediction interval was estimated for each comparison. Nimbulkar et al. [25] and Gera et al. [26] were excluded from the quantitative synthesis because their behaviour data were incomplete or not directly comparable with the prespecified first‐visit outcome. All tests were two‐sided, and *p* < 0.05 was considered statistically significant. Funnel plots and regression‐based tests for small‐study effects were not performed because fewer than 10 studies contributed to each comparison.

DC outcomes were synthesised narratively because the studies did not report a common outcome construct. Measures included caries presence or absence, ordinal severity categories, mean dft, deft, dmft or DMFT scores, and categorical caries‐risk assessments. Studies also differed in participant age, dentition, recruitment setting, diagnostic criteria and adjustment for dietary, socioeconomic and oral‐hygiene factors; a single pooled estimate was therefore considered clinically inappropriate. Analyses were performed using Python 3.13.5, NumPy 2.3.5 and SciPy 1.17.0. Forest plots were prepared using Matplotlib 3.10.8.

The certainty of evidence for each behavioural comparison was assessed using the GRADE framework [27] and presented in a summary‐of‐findings table prepared with GRADEpro GDT (McMaster University and Evidence Prime, 2023). Because all included studies were observational, each body of evidence began at a low certainty. Evidence was then assessed for risk of bias, inconsistency, indirectness, imprecision and publication bias, together with criteria that could justify rating up. The large observed associations were not rated up because the pooled estimates were unadjusted and remained vulnerable to confounding, sparse data, heterogeneity and imprecision.

## 3. Results

### 3.1. Study Selection

**Figure 1:**
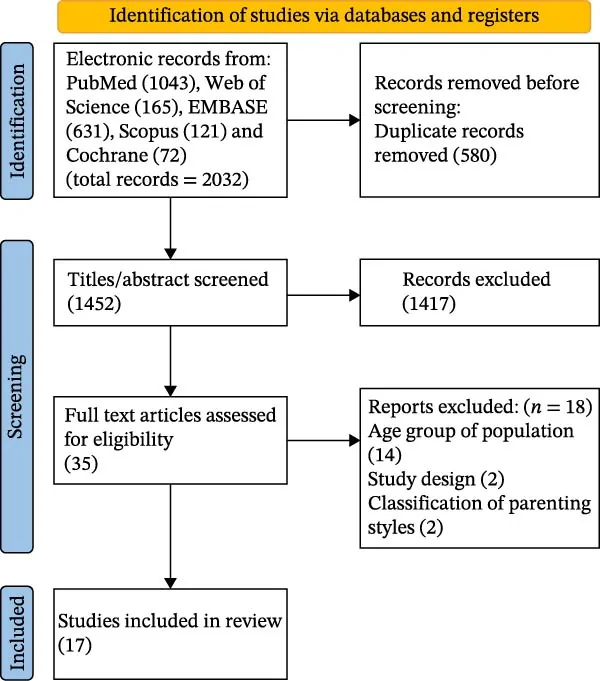
PRISMA flow diagram.

A total of 2032 records were identified through database searches: PubMed (*n* = 1043), Web of Science (*n* = 165), Scopus (*n* = 121), Embase (*n* = 631) and the Cochrane Library (*n* = 72). After removal of 580 duplicates, 1452 records underwent title and abstract screening; 1417 were excluded as clinical case reports, commentaries, reviews or records unrelated to the review question. The full texts of 35 articles were assessed, and 18 were excluded because they did not meet the prespecified age, study‐design or parenting‐style classification criteria. Seventeen studies were included in the systematic review and data extraction. No additional eligible publications were identified from the reference list screening. The study selection process is shown in Figure 1.

### 3.2. Study Characteristics

The 17 included studies were conducted in India (*n* = 7), Iran and Egypt (*n* = 2 each), and Brazil, Indonesia, Saudi Arabia, Singapore, South Korea and the United States (*n* = 1 each). All employed a cross‐sectional design. Four studies were published in 2020 (23.5%); two each in 2015, 2021, 2022, 2023 and 2025 (11.8% each); and one each in 2018, 2019 and 2024 (5.9% each). Thirteen studies (76.5%) restricted their samples to children aged 6 years or younger. Thirteen studies (76.5%) explicitly used the Parenting Styles and Dimensions Questionnaire (PSDQ) or a translated or adapted version, including El‐Mahdy et al. [28]. Aminabadi et al. [29] used the Primary Caregivers’ Practices Report (PCPR), Misrohmasari and Prihatiningrum [30] used a 25‐item parenting‐style questionnaire whose source instrument was not specified, and Amirabadi et al. [31] used the 30‐item Baumrind Parenting Style Questionnaire. Gera et al. [26] described using a parenting‐style dimensions questionnaire, but the specific form was not reported in sufficient detail to classify it confidently as a PSDQ version. Arrepia et al. [32] used the term ‘democratic’ for the category corresponding to authoritative parenting, while Alagla et al. [33] reported no participants with an authoritarian PS. Twelve studies assessed dental behaviour or cooperation, 12 assessed DC, and seven assessed both outcomes. The characteristics and principal findings of the included studies are summarised in Table 1. The quantitative synthesis of dental behaviour and narrative synthesis of DC are presented in Sections 3.4 and 3.5, respectively.

**Table 1:** Descriptive findings of the included studies.

Study	Design	Country	Sample size	Age (years)	Parenting‐style measure	Parenting‐style distribution	Child behaviour/cooperation	Dental‐caries outcome
Howenstein et al. [1]	Cross‐sectional	USA	132 enrolled; 131 in the three‐style analysis	3–6	32‐item short‐form PSDQ	Authoritative 87/132 (65.9%); authoritarian 11/132 (8.3%); permissive 33/132 (25.0%); mixed authoritative/permissive 1/132 (0.8%).	Positive/definitely positive: authoritative 81/87 (93.1%), authoritarian 5/11 (45.5%), permissive 19/33 (57.6%); *p* < 0.001.	Caries present: authoritative 17/87 (19.5%), authoritarian 10/11 (90.9%), permissive 32/33 (97.0%); *p* < 0.001.
Aminabadi et al. [29]	Cross‐sectional	Iran	288	4–6	Primary Caregivers’ Practices Report (PCPR)	Authoritative 138/288 (47.9%); authoritarian 42/288 (14.6%); permissive 108/288 (37.5%).	Authoritative scores correlated positively with positive behaviour and negatively with anxiety; permissive scores correlated with negative behaviour and anxiety (all *p* < 0.05).	Not assessed.
Nayyar et al. [34]	Cross‐sectional	India	200	3–6	PSDQ	Authoritative 115/200 (57.5%); authoritarian 37/200 (18.5%); permissive 48/200 (24.0%).	Positive behaviour: authoritative 88/115 (76.5%), authoritarian 14/37 (37.8%), permissive 13/48 (27.1%); *p* = 0.0001.	Caries present: authoritative 9/115 (7.8%), authoritarian 32/37 (86.5%), permissive 45/48 (93.8%); *p* = 0.0001.
Arrepia et al. [32]	Cross‐sectional	Brazil	67	2–6	Brazilian PSDQ	Democratic (authoritative equivalent) 33/67 (49.3%); authoritarian 4/67 (6.0%); permissive 30/67 (44.8%).	No significant association with behaviour during the initial dental visit.	No significant association with caries prevalence.
Lee et al. [35]	Cross‐sectional	South Korea	158 respondents (of 353 dyads)	3–6	Translated PSDQ	Authoritative 151/158 (95.6%); authoritarian 6/158 (3.8%); permissive 1/158 (0.6%).	Not assessed.	No significant categorical difference. The authoritative group had the lowest mean dft, and higher authoritative‐domain scores were associated with lower dft; interpretation is limited by *n* = 6 and *n* = 1 in the other groups.
Sabbarwal et al. [36]	Cross‐sectional	India	300	3–5	21‐item, locally adapted PSDQ	Authoritative 192/300 (64.0%); authoritarian 30/300 (10.0%); permissive 78/300 (26.0%).	Not assessed.	Mean dmft: authoritative 1.51 ± 1.94, authoritarian 2.10 ± 1.67, permissive 4.25 ± 3.43; *p* < 0.001.
Viswanath et al. [14]	Cross‐sectional	India	315	3–7	30‐item Tamil‐translated PSDQ	Authoritative 240/315 (76.2%); authoritarian 30/315 (9.5%); permissive 45/315 (14.3%).	Positive behaviour: 225/240 (93.8%), 14/30 (46.7%), and 17/45 (37.8%), respectively. Versus authoritative: authoritarian OR 2.232 (95% CI 0.842–5.919); permissive OR 4.354 (2.992–6.337) for negative behaviour.	Caries‐free/low/high: authoritative 56%/39%/5%; authoritarian 47%/53%/0%; permissive 13%/42%/45%. Reported permissive‐vs‐authoritative ordinal OR 3.752 (95% CI 0.597–5.624); this CI includes 1. The reported authoritarian OR/CI are internally inconsistent.
Nimbulkar et al. [25]	Cross‐sectional	India	77	4–12	32‐item Marathi‐translated PSDQ	Authoritative 29/77 (37.7%); authoritarian 22/77 (28.6%); permissive 23/77 (29.9%). These counts total 74; three participants were not classified/reported.	Counts (definitely positive; positive): authoritative 2; 14, authoritarian 1; 10, permissive 1; 10. The report does not provide a clear association *p* value; no reliable style–behaviour association can be concluded.	Not assessed.
Quek et al. [37]	Cross‐sectional	Singapore	389	4–6	PSDQ	Authoritative 370/389 (95.1%); authoritarian 4/389 (1.0%); permissive 15/389 (3.9%).	Dental‐operatory behaviour/cooperation was not assessed.	Parenting style was not associated with dmft (*p* = 0.72) or plaque index (*p* = 0.34). The very small authoritarian group limited categorical comparisons.
Alagla et al. [33]	Cross‐sectional	Saudi Arabia	282	3–6	PSDQ	Authoritative 265/282 (94.0%); authoritarian 0/282 (0%); permissive 17/282 (6.0%).	Positive/definitely positive behaviour occurred in 205/282 (72.7%). Parenting style was associated with parental dental anxiety (*p* = 0.02) but not significantly with child behaviour.	Not assessed.
Khattab et al. [38]	Cross‐sectional	Egypt	105	3–6	32‐item PSDQ	Group sizes were not reported.	Reported mean behaviour scores: authoritative 2.03 ± 0.75, authoritarian 3.35 ± 0.49, permissive 3.50 ± 0.61. The article interprets the lower authoritative score as more positive behaviour.	Mean dmf: authoritative 2.26 ± 1.31, authoritarian 3.94 ± 1.20, permissive 4.08 ± 1.61; the article reports a significant difference.
Misrohmasari and Prihatiningrum [30]	Cross‐sectional	Indonesia	269	4–6	25‐item parenting‐style questionnaire; pilot‐tested for validity/reliability, but the source instrument was not specified	Authoritative 220/269 (81.8%); authoritarian 26/269 (9.7%); permissive 23/269 (8.6%).	Not assessed.	Mean def‐t: authoritative 10.14, authoritarian 10.42, permissive 8.74; no significant difference (*p* = 0.473).
Subramani et al. [39]	Cross‐sectional	India	270	12	PSDQ	Authoritative 123/270 (45.6%); authoritarian 73/270 (27.0%); permissive 74/270 (27.4%).	Not assessed.	Mean DMFT±reported dispersion: authoritative 1.66 ± 0.12, authoritarian 1.42 ± 0.48, permissive 4.50 ± 2.14. Values are reproduced as reported; the analysis/reporting warrants caution.
Shalini et al. [40]	Cross‐sectional	India	1216	3–6	PSDQ	Authoritative 853/1216 (70.1%); authoritarian 248/1216 (20.4%); permissive 115/1216 (9.5%).	Permissive parenting showed more negative behaviour. Versus permissive, authoritative parenting had 4.102‐fold higher odds of definitely positive behaviour (*p* = 0.004).	Caries present: authoritative 41.6%, authoritarian 55.2%, permissive 47.8%. Authoritarian vs permissive OR 1.45 for caries (*p* = 0.106); positive behaviour was associated with 2.406‐fold higher odds of being caries‐free (*p* < 0.001).
El‐Mahdy et al. [28]	Cross‐sectional	Egypt	180	3–6	PSDQ	Authoritative 121/180 (67.2%); authoritarian 6/180 (3.3%); permissive 53/180 (29.4%).	Positive/definitely positive: authoritative 97/121 (80.2%), authoritarian 4/6 (66.7%), permissive 48/53 (90.6%). The article reports an influence of parenting style on behaviour.	Mean dmf: authoritative 8.31 ± 4.66, authoritarian 4.50 ± 2.88, permissive 7.18 ± 4.96; no significant difference (*p* = 0.07).
Amirabadi et al. [31]	Cross‐sectional	Iran	88	4–6	30‐item Baumrind Parenting Style Questionnaire	Authoritative 75/88 (85.2%); authoritarian 7/88 (8.0%); permissive 6/88 (6.8%).	Positive/completely positive cooperation: authoritative 58/75 (77.3%); authoritarian 0/7; permissive 0/6 (Monte Carlo *χ* ^2^, *p* < 0.001).	Not assessed.
Gera et al. [26]	Cross‐sectional	India	120	4–8	Parenting Styles and Dimensions Questionnaire; exact version and validation details were insufficiently reported	Authoritative 40; authoritarian 40; permissive 40. These were constructed equal groups and should not be interpreted as prevalence estimates.	Positive/definitely positive: authoritative 24/40 (60.0%), authoritarian 13/40 (32.5%), permissive 15/40 (37.5%); *χ* ^2^ = 9.45, *p* = 0.15 for the full four‐category behaviour distribution.	Not assessed.

Note: dft/deft/dmft, primary‐tooth caries indices; DMFT, permanent‐tooth caries index.

Abbreviations: CI, confidence interval; OR, odds ratio; PCPR, primary caregivers’ practices report; PSDQ, parenting styles and dimensions questionnaire.

### 3.3. Risk of Bias

**Figure 2:**
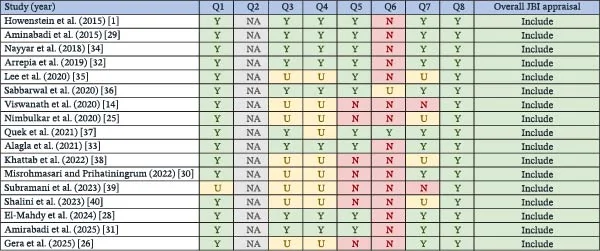
Risk of bias assessment of included studies using the JBI critical appraisal tool for cross‐sectional studies. *Note:* Y = Yes (green); N = No (red); U = Unclear (pale yellow); NA = Not applicable (grey). Colours are visual aids only and are not scores or weights. JBI domains: Q1, clarity of sample inclusion criteria; Q2, objective/standard measurement of an eligibility condition; Q3, validity and reliability of exposure measurement; Q4, validity and reliability of outcome measurement; Q5, identification of confounders; Q6, strategies addressing confounding; Q7, appropriateness of statistical analysis; Q8, detailed description of participants and setting. Appraisal levels: Q1, Q3, Q5 and Q8 are study‐level judgements; Q2, Q4 and Q6 are outcome‐level judgements; Q7 is a result‐level judgement. Q2 decision: Q2 was rated NA for all studies because eligibility was not conditional on participants having the review outcomes (dental behaviour/cooperation or dental caries); those outcomes were measured after enrolment. Overall appraisal: “Include” records the review team’s decision to retain the study. It is not an overall low‐risk rating and does not override the individual domain judgements.

The revised JBI critical appraisal tool identified adequate reporting of eligibility criteria, participants and study settings in most studies. Concerns remained regarding the validity and reliability of exposure or outcome measurement in several reports. Confounding was the principal limitation: although potential confounders were often identified, only one study adequately reported a strategy to address them. Statistical analyses were also limited by inadequate adjustment, sparse or imbalanced parenting‐style groups, and, in some studies, inappropriate handling of categorical data. All 17 studies received an overall JBI decision of include and were retained; this decision does not imply an absence of risk of bias. Item‐level judgements are presented in Figure 2.

### 3.4. Quantitative Synthesis of Child Dental Behaviour

#### 3.4.1. Authoritative Versus Permissive Parenting

Seven studies involving 1168 children contributed to this comparison. Positive behaviour was reported in 772 of 936 children with authoritative parents and 131 of 232 children with permissive parents. Authoritative parenting was associated with higher odds of positive dental behaviour (OR 4.77, 95% CI 1.10–20.76; *p* = 0.041; Figure 3a). Heterogeneity was considerable (*Q* = 50.21, *df* = 6, *p* < 0.001; *I* ^2^ = 88.1%; *τ* ^2^ = 2.072), and the 95% prediction interval was 0.09–262.96. Thus, although the pooled CI narrowly excluded the null, the expected association varied markedly across settings.

Figure 3Random‐effects meta‐analyses of parenting style and positive dental behaviour during the first dental visit: (a) Authoritative versus permissive parenting. (b) Authoritative versus authoritarian parenting. and (c) Permissive versus authoritarian parenting. Odds ratios were pooled using generic inverse‐variance models with REML estimation and modified Hartung–Knapp 95% CIs. Squares are proportional to random‐effects weights, horizontal lines show study‐specific 95% CIs, diamonds show pooled estimates, dashed red lines show 95% prediction intervals, and the vertical line indicates OR = 1. A continuity correction of 0.5 was added to all four cells for comparisons containing a single zero cell. (Amirabadi et al. was excluded from panel (c) because both comparison groups had zero positive events).

**Figure:**
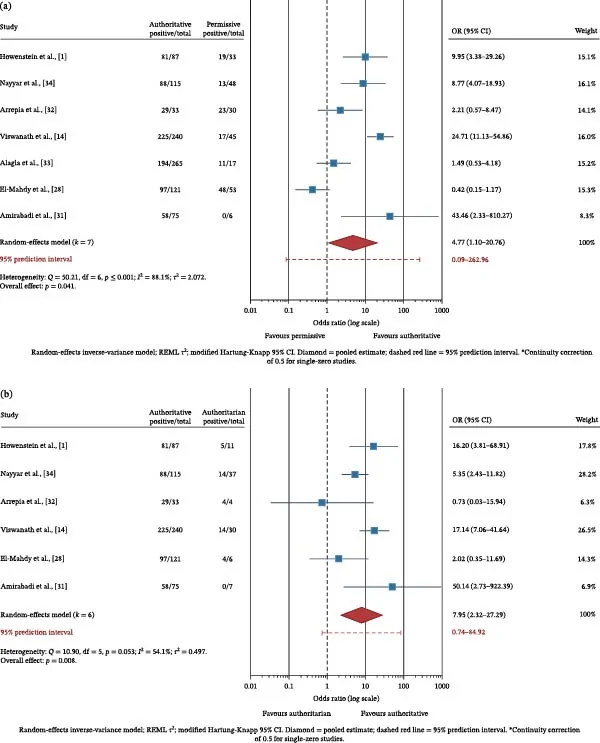

**Figure:**
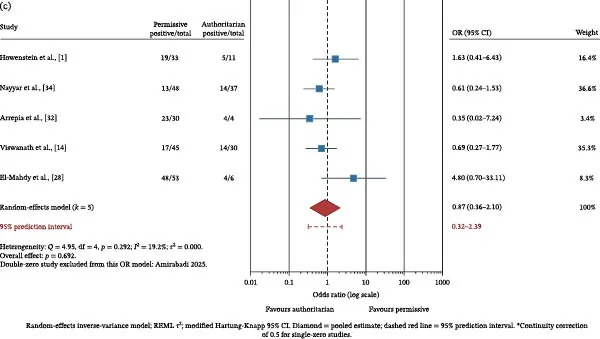

#### 3.4.2. Authoritative Versus Authoritarian Parenting

Six studies comprising 766 children contributed to this comparison. Positive behaviour was reported in 578 of 671 children with authoritative parents and 41 of 95 children with authoritarian parents. Authoritative parenting was associated with higher odds of positive dental behaviour (OR 7.95, 95% CI 2.32–27.29; *p* = 0.008; Figure 3b). Heterogeneity was moderate (*Q* = 10.90, *df* = 5, *p* = 0.053; *I* ^2^ = 54.1%; *τ* ^2^ = 0.497), and the 95% prediction interval was 0.74–84.92, which crossed the null.

#### 3.4.3. Permissive Versus Authoritarian Parenting

Five studies involving 297 children contributed to the relative‐effect model after exclusion of the Amirabadi et al. [31] double‐zero comparison. Positive behaviour was reported in 120 of 209 children with permissive parents and 41 of 88 children with authoritarian parents. No clear difference was demonstrated (OR 0.87, 95% CI 0.36–2.10; *p* = 0.692; Figure 3c). Heterogeneity was low (*Q* = 4.95, *df* = 4, *p* = 0.292; *I* ^2^ = 19.2%; *τ* ^2^ = 0), and the 95% prediction interval was 0.32–2.39. This result should not be interpreted as evidence of equivalence.

### 3.5. Narrative Synthesis of DC Outcomes

Twelve studies assessed DC using different indices and outcome definitions; therefore, meta‐analysis was not considered appropriate [1, 14, 28, 30, 32, 34–40]. Howenstein et al. [1] reported caries in 19.5% of children of authoritative parents, compared with 90.9% and 97.0% in the authoritarian and permissive groups, respectively (*p* < 0.001). Nayyar et al. [34] reported a similar pattern (7.8%, 86.5% and 93.8%; *p* = 0.0001). Sabbarwal et al. [36] found mean dmft scores of 1.51, 2.10 and 4.25, whereas Khattab et al. [38] reported mean dmf scores of 2.26, 3.94 and 4.08 in the authoritative, authoritarian and permissive groups, respectively; both studies reported significant differences. Viswanath et al. [14] and Subramani et al. [39] also reported higher caries levels in the permissive group, although limitations in their statistical reporting require cautious interpretation. Shalini et al. [40] found caries in 41.6%, 55.2% and 47.8% of the authoritative, authoritarian and permissive groups, respectively; however, the reported comparison between the authoritarian and permissive groups was not significant (*p* = 0.106). Arrepia et al. [32] found no significant association between PS and caries prevalence. Lee et al. [35] reported mixed findings, with no significant categorical difference but lower mean dft scores in the authoritative group. Quek et al. [37] found no association with dmft (*p* = 0.72), while Misrohmasari and Prihatiningrum [30] reported no significant difference in mean def‐t scores (*p* = 0.473). El‐Mahdy et al. [28] also found no significant between‐group difference in mean dmf scores (*p* = 0.07). Overall, some studies suggested lower caries occurrence or severity among children of authoritative parents, particularly compared with permissive parents, but the findings were inconsistent. Differences in caries measures, sample characteristics, group sizes and adjustment for confounding factors limit direct comparisons. The available cross‐sectional evidence therefore suggests a possible association but does not establish causality.

### 3.6. GRADE Analysis

All three bodies of evidence began at low certainty because the included studies were observational and were downgraded to very low certainty because of serious risk of bias, particularly unadjusted estimates and inadequate control of confounding. Authoritative versus permissive parenting was further limited by very serious inconsistency (*I* ^2^ = 88.1%; 95% prediction interval 0.09–262.96) and serious imprecision. Authoritative versus authoritarian parenting was limited by serious inconsistency (*I* ^2^ = 54.1%; 95% prediction interval 0.74–84.92), whereas permissive versus authoritarian parenting had very serious imprecision because its CI included potentially important effects in both directions. No serious indirectness was identified. No downgrade was applied for publication bias, although it could not be formally assessed because fewer than 10 studies contributed to each comparison. To summarise, GRADE indicated very low‐certainty evidence for all three comparisons (Table 2).

**Table 2:** GRADE certainty assessment and summary of findings.

Certainty assessment							Summary of findings				
Participants (studies) Follow‐up	Risk of bias	Inconsistency	Indirectness	Imprecision	Publication bias	Overall certainty of evidence	Study event rates (%)		Relative effect (95% CI)	Anticipated absolute effects	
							Positive behaviour with comparator style	Positive behaviour with index style		Risk with comparator style	Risk difference with index style
Authoritative vs. permissive											
1168 (7 non‐randomised studies) Follow‐up: N/A	Serious^a^	Very serious^b^	Not serious	Serious^c^	Not assessed^d^	⊕◯◯◯ Very low	131/232 (56.5%)	772/936 (82.5%)	OR 4.77 (1.10 to 20.76)	565 per 1000	296 more per 1000 (from 23 more to 400 more)
Authoritative vs. authoritarian											
766 (6 non‐randomised studies) Follow‐up: N/A	Serious^a^	Serious^e^	Not serious	Not serious	Not assessed^d^	⊕◯◯◯ Very low	41/95 (43.2%)	578/671 (86.1%)	OR 7.95 (2.32 to 27.29)	432 per 1000	426 more per 1000 (from 206 more to 522 more)
Permissive vs. authoritarian											
297 (5 non‐randomised studies) Follow‐up: N/A	Serious^a^	Not serious	Not serious	Very serious^f^	Not assessed^d^	⊕◯◯◯ Very low	41/88 (46.6%)	120/209 (57.4%)	OR 0.87 (0.36 to 2.10)	466 per 1000	34 fewer per 1000 (from 227 fewer to 181 more)

*Note:* The comparator style is the second style named in each comparison; the index style is the first. Baseline risk is the aggregated comparator event rate. Observational evidence began at low certainty and was further downgraded according to the domain judgements shown.

^a^Serious risk of bias: cross‐sectional studies, unadjusted pooled estimates and inadequate control of confounding.

^b^Very serious inconsistency: *I* ^2^ = 88.1%; 95% prediction interval 0.09–262.96.

^c^Serious imprecision: the confidence interval ranges from a potentially trivial to a very large benefit.

^d^No downgrade for publication bias was applied; formal assessment was not possible because fewer than 10 studies contributed to each comparison.

^e^Serious inconsistency: *I* ^2^ = 54.1%; 95% prediction interval 0.74–84.92 crosses no effect.

^f^Very serious imprecision: the confidence interval includes important effects favouring either parenting style.

## 4. Discussion

The aim of the current systematic review was to assess the association between different PSs, children’s behaviour in the dental operatory, and caries experience. New‐age parenting influences the child health and cognitive outcomes to a greater extent. The growing awareness of this influence has impelled an increase in research focus, especially in the field of paediatric dentistry, where child’s behaviour and their oral health habits are strongly shaped by the PSs. Most of the included studies examined children under 7 years of age, reflecting the high influence of PS during the early formative years in the life of the child. The most popular classification of PSs was given by Baumrind [7], describing parents as mostly authoritative, authoritarian and permissive. Later, neglectful/uninvolved PS was added by Maccoby and Martin [41].

The literature search period was chosen after conducting an initial literature search, and it was inferred that parental approaches have mostly evolved over the past decade owing to new age parenting trends due to dynamic family structures. Consequently, more recent studies are likely to better represent present‐day parent–child interactions and their influence on children’s behaviour and oral health. Across 17 cross‐sectional studies, authoritative parenting was the most frequently reported style. In the primary meta‐analysis, authoritative parenting was associated with higher odds of positive dental behaviour than permissive or authoritarian parenting, whereas no clear difference was found between permissive and authoritarian parenting. These estimates require cautious interpretation. The studies were observational, the pooled ORs were based on unadjusted event counts, and the prediction intervals for both authoritative comparisons crossed the null. The GRADE certainty was therefore very low. The present review integrates behavioural and clinical oral health outcomes, but it treats them according to their evidential structure. Dental behaviour was pooled only when first‐visit or no‐previous‐treatment data could be mapped to a common positive‐versus‐negative outcome. DC was synthesised narratively because the reported constructs and scales were not clinically interchangeable. This approach improved interpretability but necessarily reduced the number of studies contributing to each pooled comparison.

PSDQ was used in 13 of the 17 studies, while the remaining studies used the PCPR, the Baumrind Parenting Style Questionnaire or another questionnaire based on Baumrind’s classification (Table 1). This shared theoretical basis provided a broadly common framework for classifying authoritative, authoritarian and permissive parenting. It did not, however, ensure measurement equivalence across countries, languages or age groups. Cultural adaptation, self‐reporting and the relevance of some autonomy‐related items for very young children may have influenced classification and contributed to heterogeneity.

The behavioural findings are plausible: warmth, predictable limits and open communication may help a child regulate anxiety and cooperate in an unfamiliar clinical setting. Nevertheless, the pooled results do not show that PS determines behaviour. The estimates were based on unadjusted cross‐sectional data, and considerable heterogeneity and wide prediction intervals indicate that the associations may vary across settings.

Caries findings were inconsistent, and differences in outcome definitions, subgroup sizes and statistical adjustment precluded quantitative pooling. The extreme unadjusted group differences reported in some clinic‐based samples may therefore reflect selection, sparse subgroup counts or confounding as well as PS. The present evidence supports neither a causal nor a protective interpretation of DC. Several studies also examined outcomes outside the primary review question, including traumatic dental injuries, oral mucosal lesions, toothbrushing, dietary practices and socioeconomic characteristics. Sabbarwal et al. [36] reported more traumatic dental injuries and oral mucosal lesions among children of permissive parents, while Quek et al. [37] found that permissive parenting was associated with more frequent omission of bedtime toothbrushing. These secondary associations are clinically relevant but exploratory and do not establish the mechanisms linking PS with dental behaviour or disease. In comparison with previous reviews on this topic [20], the current review provides a more elaborate and contemporary synthesis of the available evidence by incorporating recently published studies up to December 2025. The inclusion of these newer studies expanded the geographical representation and increased the overall sample size, thereby strengthening the evidence base. The strengths of this review include PRISMA‐based reporting, prespecified eligibility criteria, random‐effects models with modified Hartung–Knapp inference and GRADE assessment. However, there are a few important limitations which should be taken into consideration. All included studies were cross‐sectional; pooled estimates were based on unadjusted aggregate counts; PS and behaviour measures were not applied uniformly; and some parenting‐style groups were small or contained zero events. The authoritative‐versus‐permissive comparison showed considerable heterogeneity, caries outcomes were not suitable for pooling, and publication bias could not be formally assessed because fewer than 10 studies contributed to each comparison. Language and publication period restrictions may also have omitted relevant evidence. These limitations are reflected in the very low‐certainty GRADE ratings.

Taken together, the PS should be regarded as a contextual marker rather than an isolated exposure. The association observed for authoritative parenting may partly represent more specific and modifiable behaviours, including calm communication, consistent routines, supervision and constructive limit‐setting. These pathways should be tested directly instead of attributing outcomes to a broad parenting category alone. Although most included studies employed validated instruments such as the PSDQ and standardised caries indices, variability in application or the use of non‐validated questionnaires in some contexts cannot be ruled out. Also, PSDQ is a self‐reported questionnaire which might introduce potential bias in parental responses, particularly in assessing PSs. Such heterogeneity in measurement methods may influence the strength and comparability of the reported associations between PS, child behaviour, and caries experience. It has been further agreed upon that certain questionnaire items—particularly those assessing autonomy, decision‐making, or participation in family matters in the PSDQ questionnaire may appear less directly applicable to very young children; however, as mentioned earlier as well that it stands to be the widely used tool for assessing parental style. Future research should use prospective, adequately powered multicentre studies with culturally validated parenting measures, standardised age and dental‐behaviour outcomes, complete cell counts and prespecified adjustment for child, parental and socioeconomic confounders. Longitudinal studies should assess caries incidence, while interventions should target modifiable communication, supervision and oral‐health routines rather than broad parenting categories.

### 4.1. Clinical Implications of the Review

Awareness of family communication and limit setting may help paediatric dentists plan conversations with parents and anticipate situations in which a child may need additional support. PS should not be used as a diagnostic label or as a substitute for direct assessment of the child’s anxiety, temperament, developmental level and behaviour. Clinicians can encourage calm, non‐threatening language, avoid transmission of parental dental anxiety and reinforce consistent toothbrushing and dietary routines. The very low‐certainty evidence does not justify recommending a change in PS solely to improve dental cooperation or caries outcomes.

## 5. Conclusion

Authoritative parenting was associated with higher odds of positive dental behaviour than permissive or authoritarian parenting, while no clear difference was demonstrated between permissive and authoritarian parenting. These findings are based on very low‐certainty, cross‐sectional evidence and the wide prediction intervals indicate that the association may differ substantially across settings. DC findings were inconsistent and could not be pooled. PS may be a relevant contextual factor in paediatric dental care, but causal or protective claims are not supported. Prospective studies using standardised outcomes and adequately adjusted analyses are required.

## Author Contributions

**Gauri Kalra**: conceptualisation, data curation, investigation, formal analysis, writing – original draft, writing – review and editing. **Apurva Mishra**: conceptualisation, data curation, investigation, resources, formal analysis, methodology, writing – original draft, writing – review and editing. **Kusum Bharti**: formal analysis, validation, writing – original draft, writing – review and editing. **Vijay Prakash Mathur**: conceptualisation, data curation, resources, validation, writing – review and editing, supervision.

## Funding

The authors would like to gratefully acknowledge Ajman University, United Arab Emirates, for covering the publication charges associated with this work.

## Disclosure

All authors have read and approved the final version of the manuscript. Apurva Mishra, the corresponding author and manuscript guarantor, had full access to all of the data in this study.

## Ethics Statement

Ethical approval was not required because this study was a systematic review and meta‐analysis of data from previously published studies and involved no direct participant recruitment, intervention, or identifiable individual‐level data.

## Consent

The authors have nothing to report.

## Conflicts of Interest

The authors declare no conflicts of interest.

## Supporting Information

Additional supporting information can be found online in the Supporting Information section.

## Supporting information


**Supporting Information 1** File S1: Completed preferred reporting items for systematic reviews and meta‐analyses (PRISMA) 2020 checklist.

**Supporting Information 2** File S2: Complete electronic search strategies used for each database.

## Data Availability

The data that support the findings of this study are available from the corresponding author upon reasonable request.
