# Supplementary material for: Influence of Different Parenting Styles on Child’s Behaviour in a Dental Operatory and Dental Caries Experience: A Systematic Review and Meta‐Analysis
Source: Int J Dent. 2026 Sep 15;2026:9390631. doi: 10.1155/ijod/9390631 (PMC13575644; doi:10.1155/ijod/9390631)
Supplement: Supplementary file 1 [file IJOD-2026-9390631-s001.docx]

**PubMed search: 1043**

((("parent child relations"[MeSH Terms] OR "parenting style"[Title/Abstract] OR "parental styles"[Title/Abstract] OR "authoritative parenting"[Title/Abstract] OR "authoritarian parenting"[Title/Abstract] OR "neglectful parenting"[Title/Abstract] OR ("parenting styles"[Title/Abstract] AND "dimensions questionnaire"[Title/Abstract])) AND (("clinical study"[Publication Type] OR "observational study"[Publication Type]) AND 2014/01/01:2025/12/31[Date - Publication]) AND (("preschool"[Title/Abstract] OR "child"[Title/Abstract] OR "children"[Title/Abstract] OR "paediatric"[Title/Abstract]) AND (("clinical study"[Publication Type] OR "observational study"[Publication Type]) AND 2014/01/01:2025/12/31[Date - Publication])) AND (("dental caries"[MeSH Major Topic] OR "tooth decay"[Title/Abstract] OR "decayed teeth"[Title/Abstract] OR "caries experience"[Title/Abstract]) AND (("clinical study"[Publication Type] OR "observational study"[Publication Type]) AND 2014/01/01:2025/12/31[Date - Publication]))) OR (("child behavior"[MeSH Major Topic] OR "dental fear"[Title/Abstract] OR "frankl behavior rating"[Title/Abstract] OR "cooperative behaviour"[Title/Abstract] OR "dental anxiety"[Title/Abstract]) AND (("clinical study"[Publication Type] OR "observational study"[Publication Type]) AND 2014/01/01:2025/12/31[Date - Publication]))) AND ((humans[Filter]) AND (2014/1/1:2025/12/31[pdat]) AND (english[Filter]) AND (child[Filter] OR preschoolchild[Filter]))

**Cochrane: 72**

("parenting style" OR "parenting styles" OR "parental style" OR "parental styles" OR "parenting practice" OR "parenting practices" OR "authoritative parenting" OR "authoritarian parenting" OR "permissive parenting" OR "neglectful parenting" OR "uninvolved parenting" OR Baumrind OR "Parenting Styles and Dimensions Questionnaire" OR PSDQ OR "Parental Authority Questionnaire") in Title Abstract Keyword AND child* OR preschool* OR pediatric* OR paediatric in Title Abstract Keyword OR ("child behavior" OR "child behaviour" OR (child* NEAR/3 behavio*) OR "cooperative behavior" OR "cooperative behaviour" OR cooperat* OR uncooperat* OR Frankl OR "Frankl behavior rating" OR "Frankl behaviour rating" OR "dental anxiety" OR "dental fear") in Title Abstract Keyword OR ("dental visit" OR "dental visits" OR "first dental visit" OR "dental appointment" OR "dental appointments" OR "dental treatment" OR "dental setting" OR "dental clinic" OR "dental attendance") in Title Abstract Keyword OR ("dental caries" OR caries OR "tooth decay" OR "decayed teeth" OR dmft OR dmfs OR deft OR defs OR "caries experience") in Title Abstract Keyword

**Scopus: 121**

TITLE-ABS-KEY ( "parenting style" OR "parenting styles" OR "parental style" OR "parental styles" OR "parenting practice" OR "parenting practices" OR "parental rearing style" OR "parental rearing styles" OR "child rearing style" OR "child rearing styles" OR "child-rearing style" OR "child-rearing styles" OR "authoritative parenting" OR "authoritarian parenting" OR "permissive parenting" OR "neglectful parenting" OR "uninvolved parenting" OR "parent-child relationship" OR "parent-child relationships" OR "parent-child relation" OR "parent-child relations" OR Baumrind OR "Parenting Styles and Dimensions Questionnaire" OR PSDQ OR "Parental Authority Questionnaire" OR ( parent* W/2 style* ) ) AND TITLE-ABS-KEY ( child* OR preschool* OR pediatric* OR paediatric* ) AND ( TITLE-ABS-KEY ( "dental caries" OR caries OR "tooth decay" OR "decayed teeth" OR "caries experience" OR dmft OR dmfs OR deft OR defs ) OR ( TITLE-ABS-KEY ( "child behavior" OR "child behaviour" OR "cooperative behavior" OR "cooperative behaviour" OR cooperat* OR uncooperat* OR Frankl OR "Frankl behavior rating" OR "Frankl behaviour rating" OR "dental anxiety" OR "dental fear" ) AND TITLE-ABS-KEY ( "dental visit" OR "dental visits" OR "first dental visit" OR "first dental visits" OR "dental appointment" OR "dental appointments" OR "dental treatment" OR "dental treatments" OR "dental setting" OR "dental settings" OR "dental clinic" OR "dental clinics" OR "dental attendance" ) ) ) AND PUBYEAR > 2013 AND PUBYEAR < 2026

**Embase: 631**

('parenting'/exp OR 'parent child relation'/exp OR ('parenting style' OR 'parenting styles' OR 'parental style' OR 'parental styles' OR 'parenting practice' OR 'parenting practices' OR 'parenting pattern' OR 'parenting patterns' OR 'parental rearing style' OR 'parental rearing styles' OR 'child rearing style' OR 'child rearing styles' OR 'child-rearing style' OR 'child-rearing styles' OR 'authoritative parenting' OR 'authoritarian parenting' OR 'permissive parenting' OR 'neglectful parenting' OR 'uninvolved parenting' OR 'parent-child relationship' OR 'parent-child relationships' OR 'parent-child relation' OR 'parent-child relations' OR baumrind OR 'parenting styles and dimensions questionnaire' OR psdq OR 'parental authority questionnaire' OR (parent* NEAR/2 style*) OR (parent* NEAR/2 practice*)):ti,ab,kw) AND ('child'/exp OR (child* OR preschool* OR pediatric* OR paediatric*):ti,ab,kw) AND (('dental caries'/exp OR ('dental caries' OR 'early childhood caries' OR caries OR 'tooth decay' OR 'decayed tooth' OR 'decayed teeth' OR 'caries experience' OR dmft OR dmfs OR deft OR defs):ti,ab,kw) OR (('child behavior'/exp OR 'dental anxiety'/exp OR ('child behavior' OR 'child behaviour' OR 'dental behavior' OR 'dental behaviour' OR (child* NEAR/3 behavio*) OR 'cooperative behavior' OR 'cooperative behaviour' OR 'dental cooperation' OR cooperat* OR uncooperat* OR frankl OR 'frankl behavior rating' OR 'frankl behaviour rating' OR 'dental anxiety' OR 'dental fear'):ti,ab,kw) AND ('pediatric dentistry'/exp OR 'dental care'/exp OR ('dental visit' OR 'dental visits' OR 'first dental visit' OR 'first dental visits' OR 'dental appointment' OR 'dental appointments' OR 'dental treatment' OR 'dental treatments' OR 'dental setting' OR 'dental settings' OR 'dental clinic' OR 'dental clinics' OR 'dental attendance'):ti,ab,kw))) AND [2014-2025]/py

**Web of Science: 165**

TS=("parenting style" OR "parenting styles" OR "parental style" OR "parental styles" OR "parenting practice" OR "parenting practices" OR "parenting pattern" OR "parenting patterns" OR "parental rearing style" OR "parental rearing styles" OR "child rearing style" OR "child rearing styles" OR "child-rearing style" OR "child-rearing styles" OR "authoritative parenting" OR "authoritarian parenting" OR "permissive parenting" OR "neglectful parenting" OR "uninvolved parenting" OR "parent-child relationship" OR "parent-child relationships" OR "parent-child relation" OR "parent-child relations" OR Baumrind OR "Parenting Styles and Dimensions Questionnaire" OR PSDQ OR "Parental Authority Questionnaire" OR (parent* NEAR/2 style*) OR (parent* NEAR/2 practice*)) AND TS=(child* OR preschool* OR pediatric* OR paediatric*) AND (TS=("dental caries" OR "early childhood caries" OR caries OR "tooth decay" OR "decayed tooth" OR "decayed teeth" OR "caries experience" OR dmft OR dmfs OR deft OR defs) OR (TS=("child behavior" OR "child behaviour" OR "dental behavior" OR "dental behaviour" OR (child* NEAR/3 behavio*) OR "cooperative behavior" OR "cooperative behaviour" OR "dental cooperation" OR cooperat* OR uncooperat* OR Frankl OR "Frankl behavior rating" OR "Frankl behaviour rating" OR "dental anxiety" OR "dental fear") AND TS=("dental visit" OR "dental visits" OR "first dental visit" OR "first dental visits" OR "dental appointment" OR "dental appointments" OR "dental treatment" OR "dental treatments" OR "dental setting" OR "dental settings" OR "dental clinic" OR "dental clinics" OR "dental attendance" OR "pediatric dentistry" OR "paediatric dentistry"))) AND DOP=2014-01-01/2025-12-31
